# Neuroendocrine differentiation: a risk fellow in colorectal cancer

**DOI:** 10.1186/s12957-023-02952-8

**Published:** 2023-03-10

**Authors:** Yue Chen, Yu Liang, Lianqun Cao, Xinxin Dong, Deyu Sun

**Affiliations:** 1 Department of Colorectal Surgery, Cancer Hospital of China Medical University, Liaoning Cancer Hospital & Institute, No. 44 Xiaoheyan Road, Dadong District, Shenyang, 110042 Liaoning Province China; 2 Department of Radiation Oncology Gastrointestinal and Urinary and Musculoskeletal Cancer, Cancer Hospital of China Medical University, Liaoning Cancer Hospital & Institute, No. 44 Xiaoheyan Road, Dadong District, Shenyang, 110042 Liaoning Province China

**Keywords:** Colorectal cancer, Neuroendocrine differentiation, Lymph node metastasis, PI3K-Akt signaling pathway

## Abstract

**Background:**

Neuroendocrine differentiation (NED) is often found in colorectal cancer (CRC) and may have unique biological behavior, which has not been previously delineated. Here, we explore the relationship between CRC, NED, and clinicopathological factors. We also offer a preliminary explanation of the mechanism underlying the malignant biological behavior of NED in CRC.

**Methods:**

Between 2013 and 2015, 394 CRC patients who underwent radical operations were selected for analysis. The relationship between NED and clinicopathological factors was analyzed. To further clarify the pivotal role of NED in CRC, we performed bioinformatic analyses and identified genes that may be involved in NED, which were obtained from in silico data from The Cancer Genome Atlas (TCGA) database. Then, we conducted functional enrichment analyses and confirmed the critical pathways for intensive study. Moreover, we detected the expression of key proteins by immunohistochemistry and analyzed the correlation of their expression with NED.

**Results:**

The statistical analysis showed that CRC with NED was positively correlated with lymph node metastasis. Through bioinformatic analysis, we found that chromogranin A (CgA) was positively correlated with invasion and lymph node metastasis. ErbB2 and PIK3R1, which are key proteins in the PI3K-Akt signaling pathway, were closely related to NED. Furthermore, we determined that the PI3K-Akt signaling pathway likely plays a critical role in the NED of CRC.

**Conclusions:**

CRC with NED is associated with lymph node metastasis. The PI3K-Akt signaling pathway, which is closely related to CRC, may be the mechanism promoting the malignant biological behavior of CRC with NED.

## Background

Colorectal cancer (CRC) is one of the most common malignant cancers worldwide. Although its pathology is mainly adenocarcinoma, the components may differ. Adenocarcinoma with neuroendocrine differentiation (NED) is a special type of cancer. It usually contains two components: adenocarcinoma and a neuroendocrine tumor, but the main component is adenocarcinoma, with the percentage of neuroendocrine tumor cells less than 30% [1]. Neuroendocrine tumors originate from neuroendocrine cells which can secrete serotonin metabolites or polypeptide hormones. Because of these secretions, the tumor may produce changes in the biological behavior of surrounding cells [2]. Because NED is related to nerve and endocrine function, it can be diagnosed by detecting chromogranin A (CgA) and/or synaptophysin (SYP/Syn) proteins.

At present, NED has been found in many solid cancers, but research about NED in prostate cancer has been the most extensive. These studies have not only examined the poor effects of treatment and prognosis [3–5], but have also studied the mechanism of hormones secreted by NED cells on surrounding cells, which enhances their capacity for metastasis and invasion [6]. In vitro studies have shown that neuropeptides can stimulate androgen-independent growth [7] and increase the invasiveness of prostate cancer cells [8]. The mechanism may be related to several pathways, including PKA/CREB [9], PI3K/Akt [10], and others [11, 12]. In CRC, research has mainly focused on clinical prognosis, where conclusions have differed and are debatable [13–15]. Indinnimeo et al. [16] found a significant association between CgA-positivity and lymph node metastasis in human colon cancer. There are also some studies which showed that NED was associated with liver metastasis and advanced tumor events [17]. By contrast, early studies by Mori et al. [14] and Lloyd et al. [13] showed that NED did not influence patient prognosis. Therefore, the purpose of this research was to study whether NED influences the biological behavior of CRC and to conduct a preliminary exploration of the mechanisms underlying its malignant biological behavior.

In the present study, we retrospectively analyzed patients with NED. We then downloaded in silico data from The Cancer Genome Atlas (TCGA) (http://cancergenome.nih.gov) and identified the NED (±) samples involved based on CgA and/or SYP expression. We further performed bioinformatic analysis to reveal the mechanisms that may be involved in NED. Finally, we conducted immunohistochemical staining to verify our predictions.

## Methods

### Study population

A total of 394 patients with primary CRC, who underwent radical operation between 2013 and 2015 at Liaoning Cancer Hospital and Institute (Shenyang, Liaoning Province, People’s Republic of China) were included in this study. All patients were at stages I, II, or III according to the Union for International Cancer Control TNM classification system. Patients who died perioperatively or with secondary malignancy and those with distant metastases such as liver or lung before the operation were excluded. Histology specimens were evaluated by two senior pathologists, and the diagnosis of CRC with or without NED was confirmed in all patients. This study was approved by the local ethics committee of Liaoning Cancer Hospital and Institute.

### Bioinformatic analysis of CgA and SYP expression in CRC

#### Data collection and analysis

We downloaded a total of 568 CRC and 44 normal tissues from TCGA (https://portal.gdc.cancer.gov). The data included RNA-seq profiles and clinicopathological information. We further compared the expression of CgA and SYP between normal and malignant samples. The overall survival and progression-free survival of patients expressing high and low levels of the two markers were also acquired by applying the Kaplan–Meier method (“limma,” “survival,” and “survminer” packages in R software).

#### Pathological and functional enrichment analyses of CgA and SYP

To reveal the potential mechanisms of CgA and SYP, we further calculated the expression of the two markers in different pTNM stages through R software (“ggpubr” package). Then, we divided the CRC samples into two sets based on the expression of CgA and SYP. We analyzed differentially expressed genes (DEGs) with a threshold log2 fold change (FC) > 2.0 and *P* < 0.01. The top 100 DEGs were also drawn as a heatmap. Moreover, we conducted Kyoto Encyclopedia of Genes and Genomes (KEGG) pathway analyses of DEGs using R software.

### Immunohistochemistry

We selected three key proteins of the PI3K-Akt signaling pathway, including ErbB2, PIK3R1, and AKT3, to verify the pathway predictions. After dewaxing, the sections were subjected to antigen retrieval at high temperature and pressure for 3 min, washed with water, incubated with 3% hydrogen peroxide at room temperature for 10 min, washed again three times, and then placed in phosphate-buffered saline solution. We blocked the sections with 5% fetal bovine serum for 30 min, then added the first antibody for overnight incubation at 4 °C. The next day, the sections were washed three times, then incubated with a secondary antibody at room temperature for 30 min and washed three times. We then incubated the sections for 1–3 min with diaminobenzidine chromogenic solution, lightly stained the nuclei with hematoxylin, and differentiated the sections with 0.5% hydrochloric acid ethanol for 2 s. After three washes, the sections were dehydrated with graded alcohol, made transparent, and sealed with neutral gum. The antibodies used in the study were raised against CgA (dilution 1:1000, GT211407; Gene Tech, CHINA), SYP (dilution 1:1000, GT206507; Gene Tech), ErbB2 (dilution 1:1000, GT224507; Gene Tech), PIK3R1 (dilution 1:1000, ER64588; HUABIO), and AKT3 (dilution 1:1000, ER62638; HUABIO).

Based on the 2010 World Health Organization classification system, we examined two neuroendocrine biomarkers, CgA and SYP. Specimens in which CgA and/or SYP were present in 2–30% of immunoreactive cells were classified as NED [1]. All immunohistochemical sections were evaluated by two senior pathologists.

### Statistics

All statistical analyses were performed using SPSS (version 22.0; IBM, USA). Pearson’s chi-square and Fisher’s exact test were used to investigate correlations between clinicopathological factors and NED, as well as the relationship between NED and predictive proteins. Correlations with *P* values < 0.1 were entered into the next step of multivariate analysis using logistic regression. *P* values < 0.05 were considered statistically significant.

## Results

### General information

**Fig. 1:**
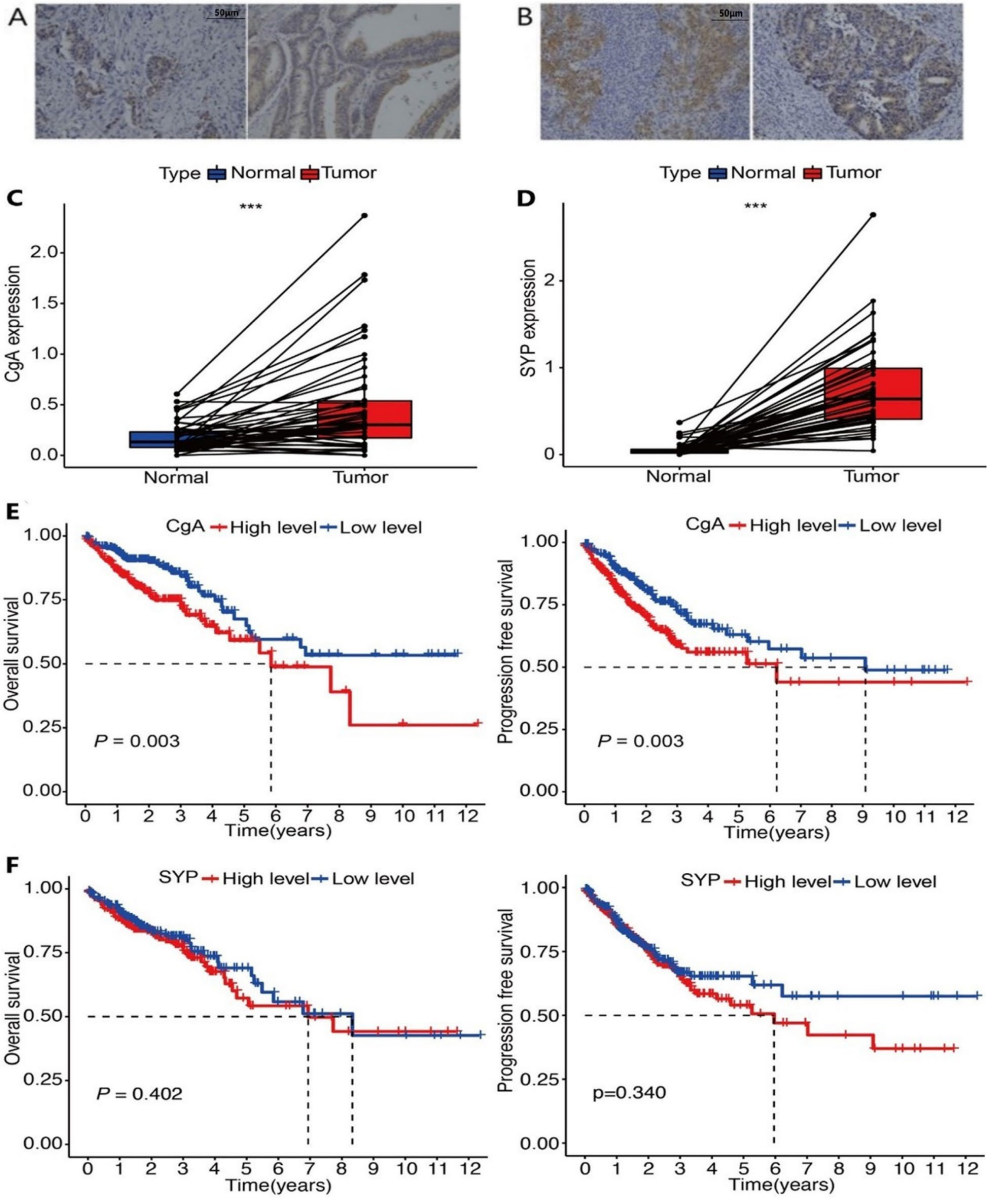
**A** Neuroendocrine tumor cells in CRC detected by immunostaining for CgA (magnification x 100). **B** Neuroendocrine tumor cells in CRC detected by immunostaining for SYP (magnification x 100). **C** Relative expression of CgA in CRC and corresponding non-cancerous tissues from TCGA. ***P < 0.001. **D** Relative expression of SYP in CRC and corresponding non-cancerous tissues from TCGA. ***P < 0.001. **E** Kaplan–Meier curve analysis of the correlation of CgA expression and overall survival or progression-free survival from TCGA. **F** Kaplan–Meier curve analysis of the correlation of SYP expression and overall survival or progression-free survival from TCGA. CgA, chromogranin A; CRC, colorectal cancer; SYP, synaptophysin; TCGA, The Cancer Genome Atlas

A total of 394 patients (234 males and 160 females) were included in the study. Among them, 55 (14.0%) were NED(+) patients including 33 CgA(+) (Fig. 1A) and 26 SYP(+) (Fig. 1B) patients, and four patients were positive for both. The mean age was 61.9 years (28–84 years). Of these patients, 43 had stage I, 121 had stage II, and 230 had stage III cancer, which included 222 cases of colon cancer and 172 cases of rectal cancer.

### Clinicopathological factors and NED

Univariate analysis showed that lymph nodes retrieved (*P* = 0.044), depth of invasion (*P* = 0.074), and lymph node metastasis (*P* = 0.020) were closely correlated with NED. Multivariate analysis showed that only lymph node metastasis (*P* = 0.022, odds ratio = 2.091) was closely correlated with NED, and NED(+) patients were more prone to lymph node metastasis, while lymph nodes retrieved and depth of invasion had no significant correlation with NED (Table 1).

**Table 1:** Correlation between clinicopathological factors and neuroendocrine differentiation

Clinicopathological factors	Number	NED		Univariate analysis	Multivariate analysis	
		+	−		*P* value	OR
Age				0.382		
<65 years	243	31	212			
≥65 years	151	24	127			
Gender				0.844		
Male	234	32	202			
Female	160	23	137			
Tumor Location				0.400		
Colon	223	34	189			
Rectum	171	21	150			
Differentiation				0.841		
Good	354	49	305			
Poor	40	6	34			
Lymph nodes retrieved				0.044^*^	0.079	
<12	125	11	114			
≥12	269	44	225			
T stage				0.074^*^	0.230	
T1+T2	51	3	48			
T3+T4	343	52	291			
N stage				0.020^*^	0.022^*^	2.091
N0	164	15	149			
N1+N2	230	40	190			
M stage				0.928		
M0	377	52	325			
M1	17	3	14			

^*^Indicated statistical significance (*P*<0.05)

### Bioinformatic analysis of CgA and SYP expression in CRC

Firstly, we compared the expression of CgA and SYP between malignant tumors and corresponding non-cancerous tissues. The results are shown in Fig. 1C, D, indicating that CgA and SYP were upregulated in CRC (*P* < 0.001). Furthermore, we performed the Kaplan-Meier curve analysis to reveal the significance of expression in overall survival and progression-free survival. As shown in Fig. 1E, the expression of CgA was negatively correlated with overall survival (*P* = 0.003) and progression-free survival (*P* = 0.003); however, there was no statistically significant difference in SYP expression (Fig. 1F).

### Pathological analyses of CgA and SYP in CRC

**Fig. 2:**
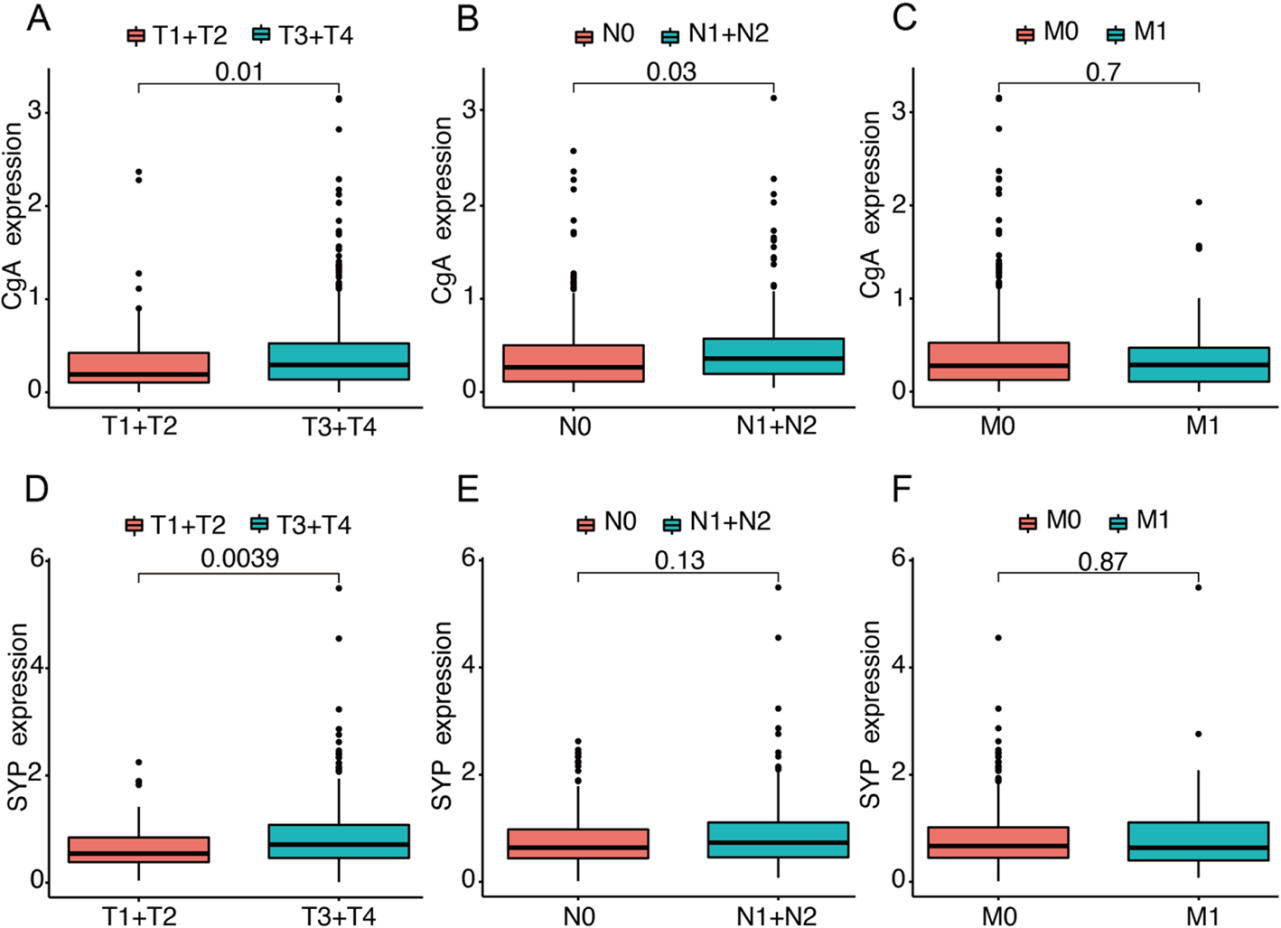
The correlation between CgA and SYP expression and pTNM stage of CRC. **A** The analyses of CgA expression in pT1+T2 stage and pT3+T4 stage. **B** The analyses of CgA expression in pN0 stage and pN1+N2 stage. **C** The analyses of CgA expression in pM0 stage and pM1 stage. **D** The analyses of SYP expression in pT1+T2 stage and pT3+T4 stage. **E** The analyses of SYP expression in pN0 stage and pN1+N2 stage. **F** The analyses of SYP expression in pM0 stage and pM1 stage. CgA, chromogranin A; CRC, colorectal cancer; SYP, synaptophysin

To further clarify the pivotal role of NED in CRC, we further analyzed the relationship between the expression of the two genes and the pTNM stage. As shown in Fig. 2A, B, the expression of CgA was positively correlated with the depth of invasion (*P* = 0.01) and lymph node metastasis (*P* = 0.03). The expression of SYP was positively correlated with pT stage (Fig. 2D, *P* = 0.0039), but was not different between pN0 and pN1+N2 stages (Fig. 2E, *P* = 0.13). We further found that CgA and SYP had no correlation with metastasis (Fig. 2C, *P* = 0.7 and Fig. 2F, *P* = 0.87).

### KEGG pathway analyses of CgA and SYP

**Fig. 3:**
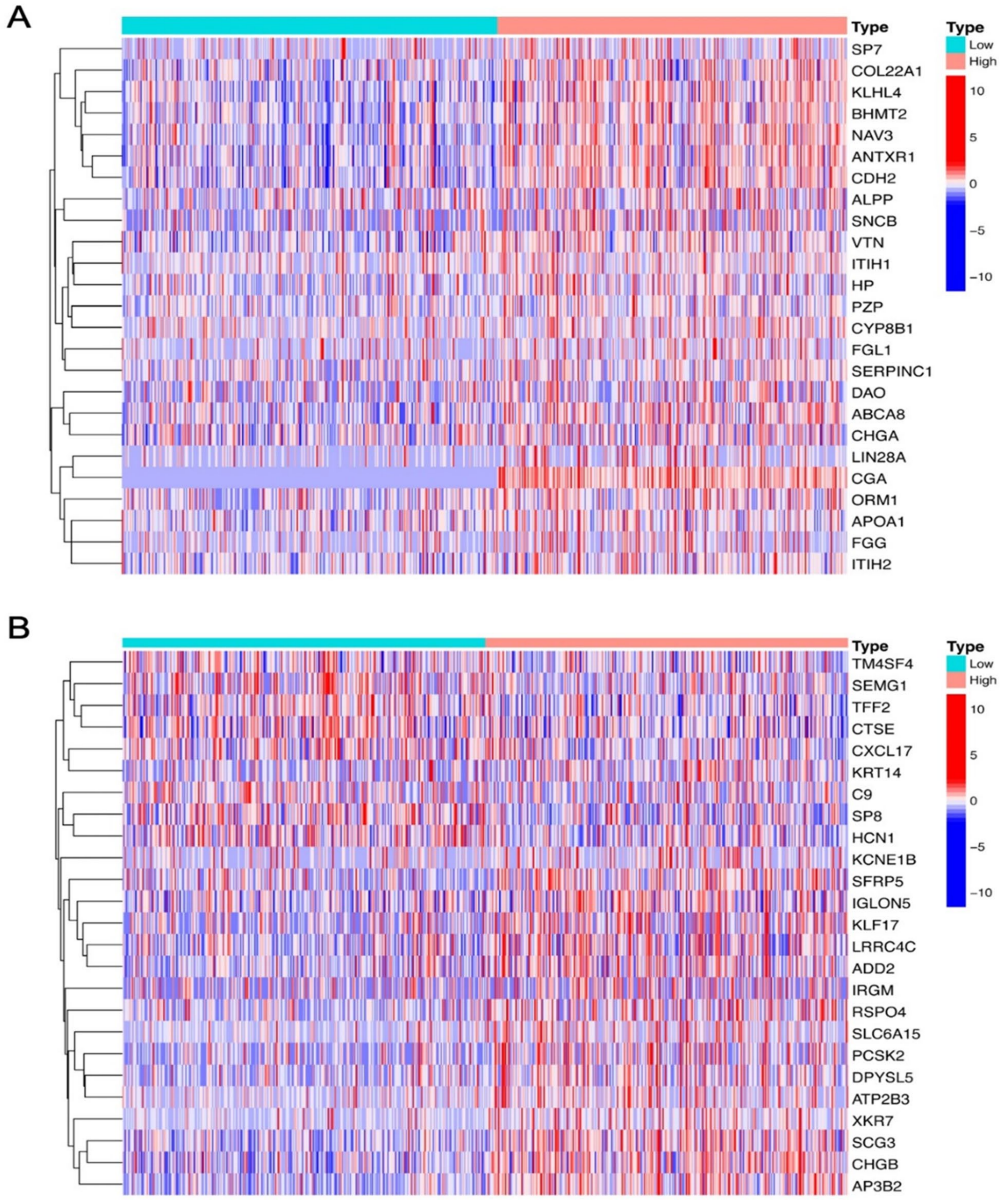
Heatmap of DEGs between upregulation and downregulation of CgA or SYP. **A** Heatmap of the top 25 DEGs between upregulation and downregulation of CgA (log2 FC > 2, *P* < 0.01). **B** Heatmap of the top 25 DEGs between upregulation and downregulation of SYP (log2 FC > 2, *P* < 0.01). The left vertical axis shows clusters of DEGs and right vertical axis represents gene names. Red represents upregulated genes and blue represents downregulated genes. CgA, chromogranin A; DEGs, differentially expressed genes; FC, fold change; SYP, synaptophysin

**Fig. 4:**
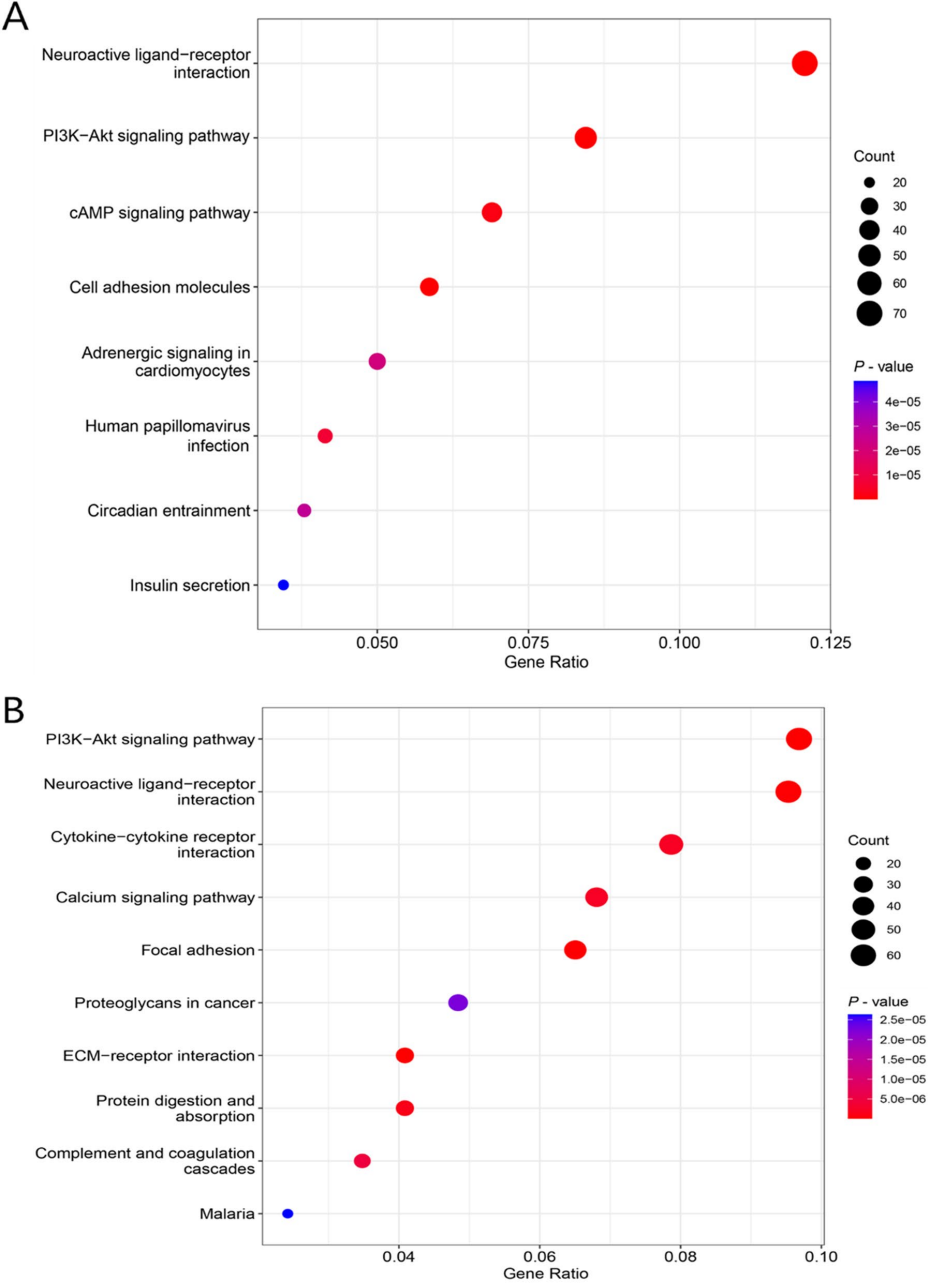
KEGG pathway enrichment network diagram for DEGs. **A** KEGG pathways enrichment analysis of CgA. **B** KEGG pathways enrichment analysis of SYP. “Count” represents the number of genes. CgA, chromogranin A; DEGs, differentially expressed genes; KEGG, Kyoto Encyclopedia of Genes and Genomes; SYP, synaptophysin

**Fig. 5:**
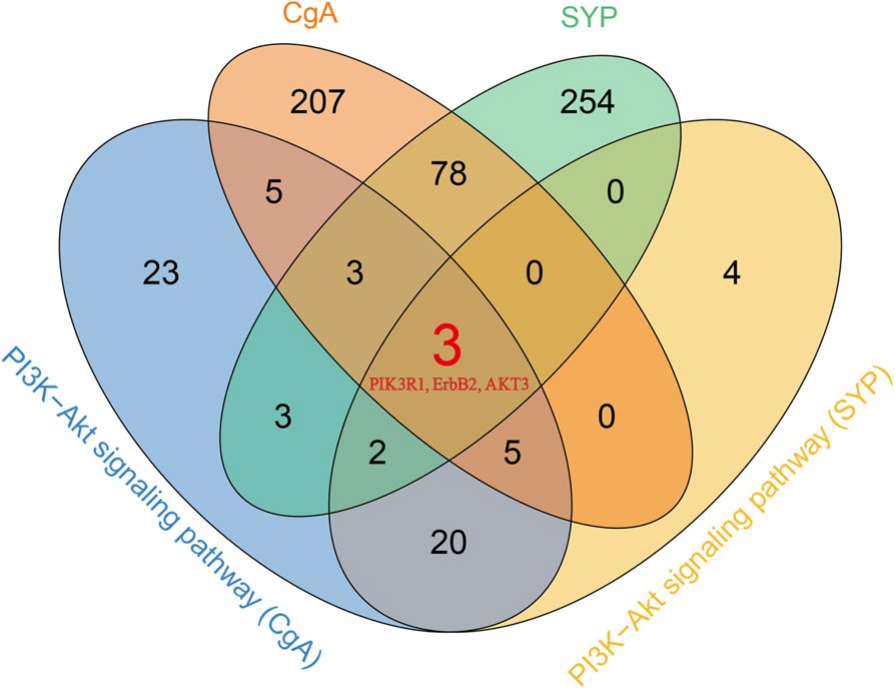
The Venn diagram of intersection analysis of consensus genes. The cluster of genes were respectively from differentially expressed genes of CgA and SYP, and the genes enriched in “PI3K-Akt signaling pathway”. CgA, chromogranin A; SYP, synaptophysin

Since CgA and SYP were closely related to clinicopathological features, this prompted us to further explore their molecular mechanisms in CRC. The two groups of DEGs were obtained based on the upregulation and downregulation of CgA and SYP, and the top 20 DEGs were mapped as heatmaps in Fig. 3A, B. Then, we performed KEGG pathway analyses of CgA and SYP. The results are shown in Fig. 4A, B and indicated that multiple cancer-related pathways were enriched. Interestingly, the “PI3K-Akt signaling pathway” and “neuroactive ligand-receptor interaction” co-occurred in both results and had the most genes involved. Because previous studies have confirmed that the PI3K-Akt signaling pathway-regulated NED in CRC, we tried to determine which genes played a role in these pathways. We performed intersection analysis on the DEGs of CgA and SYP, compared to the genes enriched in the PI3K-Akt signaling pathway. Finally, we identified three genes (PIK3R1, ErbB2, and AKT3) which intersected. The Venn diagram is shown in Fig. 5.

### Immunohistochemical staining for verification

**Fig. 6:**
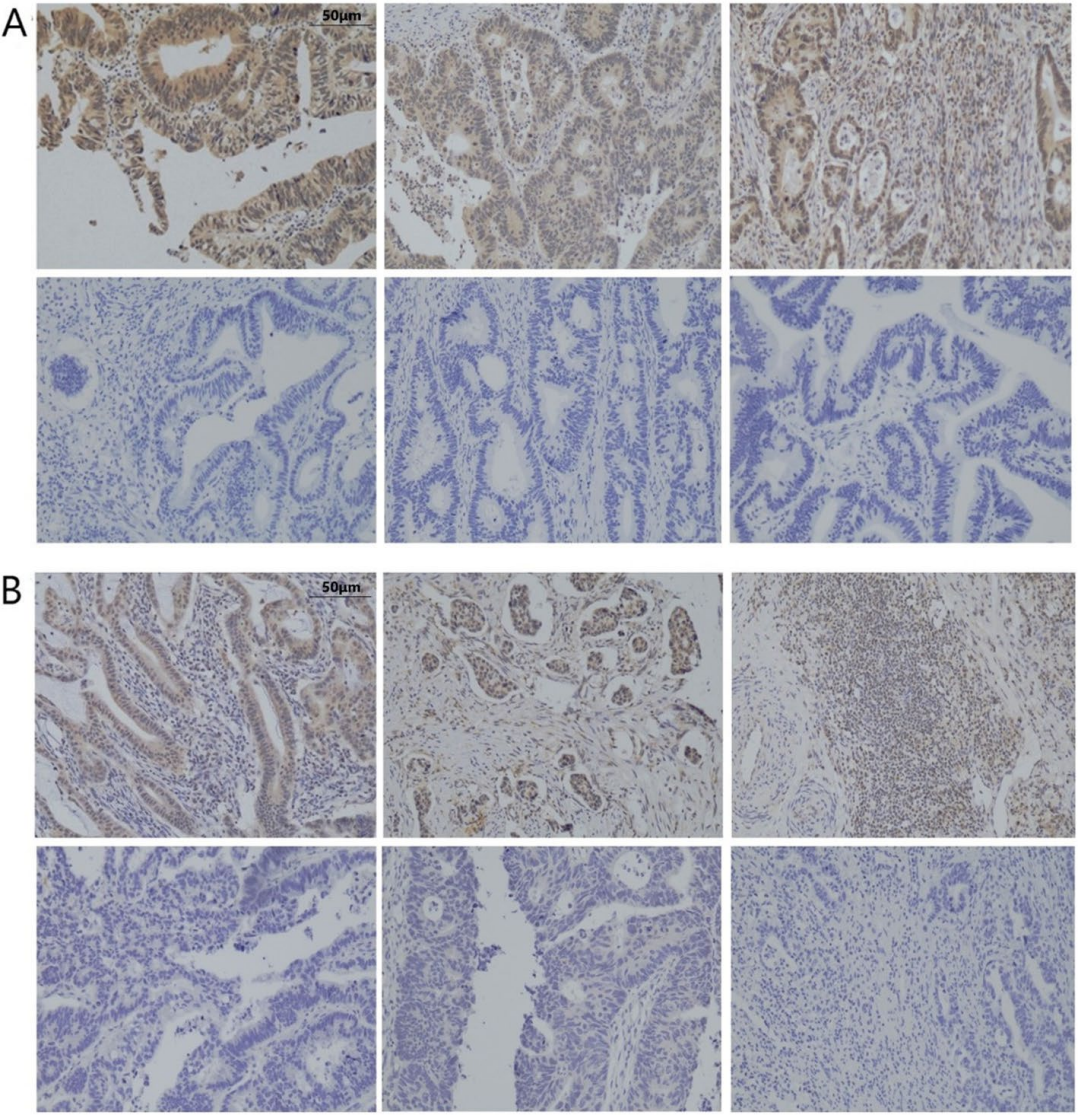
Immunohistochemical staining of key proteins in the PI3K-Akt signaling pathway. **A** ErbB2-positive and negative staining (magnification x 100). First row, positive; second row, negative. **B** PIK3R1-positive and negative staining (magnification x 100). First row, positive; second row, negative

We randomly selected 33 NED(+) patients and 34 NED(−) patients for immunohistochemistry. We selected three key proteins of the PI3K-Akt signaling pathway, including ErbB2, PIK3R1, and AKT3, for further immunohistochemical verification. After statistical analysis of these three proteins, we found that ErbB2 was positive in 14 of 33 NED(+) patients and positive in six of 34 NED(−) patients (Fig. 6A). We also found that PIK3R1 was positive in 30 of 33 NED(+)patients and positive in 19 of 34 NED(−) patients (Fig. 6B). The statistical analysis showed that ErbB2 and PIK3R1 were closely correlated with NED (Table 2).

**Table 2:** Correlation between predictive proteins and neuroendocrine differentiation

Predictive proteins	NED		Univariate analysis
	+	−	
GRB2			0.266
+	20	16	
−	13	18	
PIK3R1			0.001^*^
+	30	19	
−	3	15	
mTor			0.197^a^
+	29	33	
−	4	1	
ErbB1 (EGFR)			1.000^a^
+	32	32	
−	1	2	
ErbB2			0.027^*^
+	14	6	
−	19	28	
PPP2R1A			1.000^a^
+	32	32	
−	1	2	
AKT3			0.375
+	12	16	
−	21	18	
FSHB			0.152
+	12	7	
−	21	27	
FSHR			0.493^a^
+	1	0	
−	32	34	
TSHB			1.000^a^
+	31	32	
−	2	2	
GH			0.614^a^
+	2	1	
−	31	33	

^*^Indicated statistical significance (*P*<0.05)

^a^Fisher’s exact test

## Discussion

Adenocarcinoma with NED has been studied in CRC in many previous studies, but findings have been controversial. Some studies have shown no significant correlation between NED and prognosis [13, 18, 19], while other studies have drawn the opposite conclusion, suggesting that NED was significantly associated with the prognosis [20–22]. Furthermore, some other studies have shown that patients with NED have a higher incidence of lymph node metastasis [16] and liver metastasis [17]. In our previous study, we found that patients with NED were associated with lymph node metastasis in poorly differentiated CRC [23]. In the present study, we extended our analysis to include various differentiated adenocarcinomas and have reached the same result. We firmly believe that NED is closely related to lymph node metastasis.

**Fig. 7:**
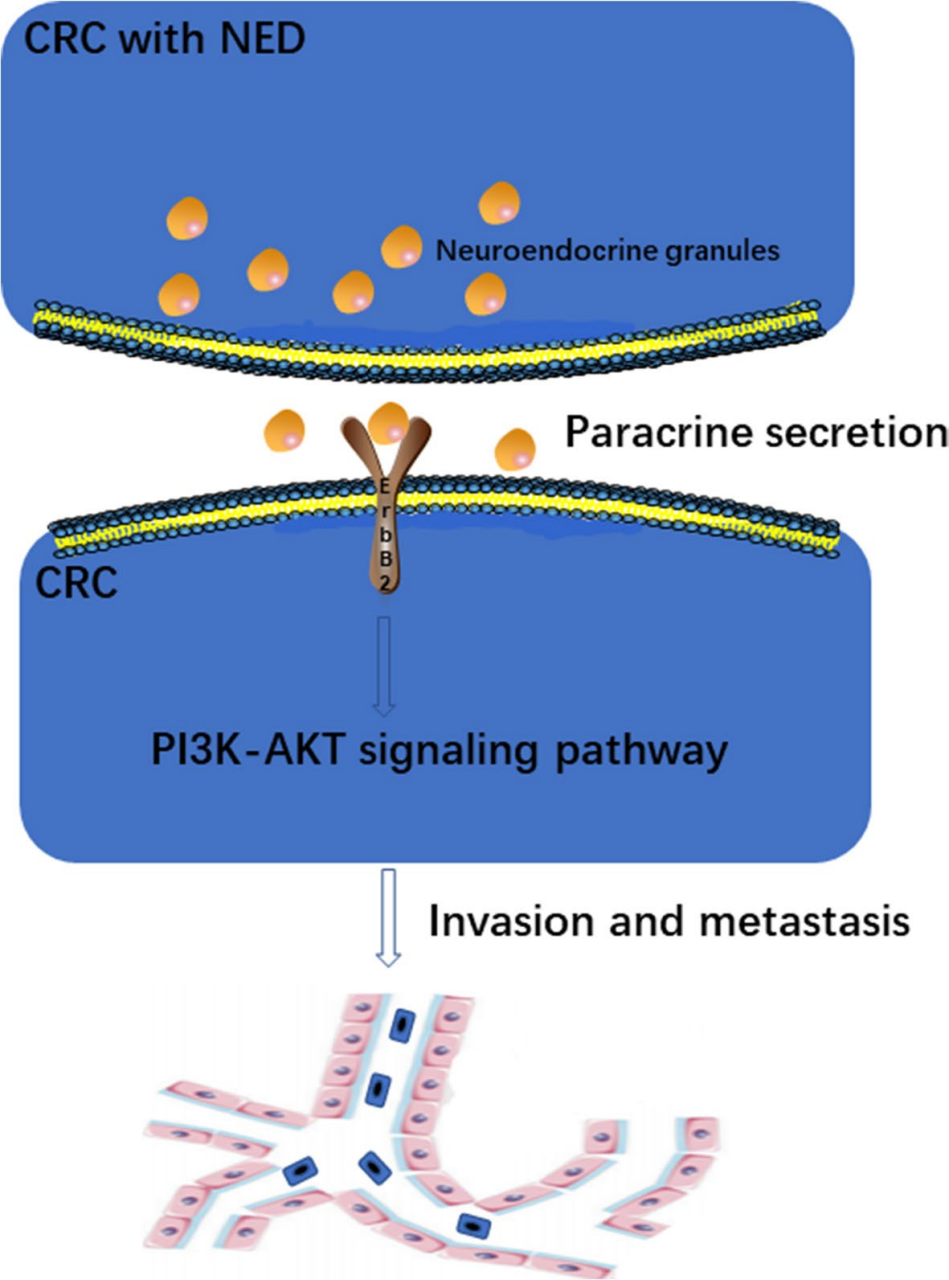
CRC with NED releases neuroendocrine granules which act on surrounding CRC cells through paracrine secretion, thus activating the PI3K-AKT signaling pathway and causing tumor invasion and metastasis. CRC, colorectal cancer; NED, neuroendocrine differentiation

What is the mechanism underlying the relationship between NED and lymph node metastasis? It is already known that NED cells secrete hormone substances in autocrine or paracrine loops and a previous study has shown that biogenic amines and polypeptide hormones play an important role in the growth regulation of normal and neoplastic intestinal epithelia [24]. Therefore, we hypothesized that NED cells may secret certain neurohormonal substances which stimulate growth and the metastatic capacity of CRC cells. In order to preliminarily confirm this hypothesis, we predicted the proteins interacting with NED using biological function analysis and verified their presence in CRC tissues by immunohistochemistry. We found ErbB2 and PIK3R1, which are key proteins in the PI3K-Akt signaling pathway, were closely correlated with NED. In previous studies, the PI3K-Akt signaling pathway was found to be closely related to the proliferation and invasion of CRC [25–28]. Therefore, we speculated that NED cells may enhance the invasion of CRC by activating the PI3K-Akt signaling pathway, so as to promote malignant biological behavior (Fig. 7). However, confirming this hypothesis will require further studies on cell function and mechanisms.

Considering the different diagnostic criteria of NED used in previous studies, the results of these studies may not be reliable [29–32]. To ensure the accuracy of our results, we re-stained all cases and use the latest recognized method for the diagnosis of NED [33, 34]. Nevertheless, there still are some limitations in our study. First, because of the nature of retrospective studies, selective bias may inevitably exist. Second, our examination of the mechanisms involved may be insufficient, as we have only conducted a preliminary exploration at the protein level, according to the results of the bioinformatic analysis. Therefore, further studies will be needed to confirm these results.

## Conclusions

In CRC, patients with NED were positively correlated with lymph node metastasis. The PI3K-Akt signaling pathway, which is closely related to the proliferation and invasion of CRC, may be the mechanism promoting the malignant biological behavior of lymph node metastasis in CRC with NED.

## Data Availability

Not applicable.

## Abbreviations

NED: Neuroendocrine differentiation
CRC: Colorectal cancer
TCGA: The Cancer Genome Atlas
CgA: Chromogranin A
SYP/Syn: Synaptophysin
DEGs: Differentially expressed genes
FC: Fold change
KEGG: Kyoto Encyclopedia of Genes and Genomes
